# Exploring the Psycho-Social Well-Being of Young Adults in Rural South Africa During the COVID-19 Lockdown: A Qualitative Study from Lonely Park, Mafikeng

**DOI:** 10.3390/ijerph22071041

**Published:** 2025-06-30

**Authors:** Dineo J. Melamu, Wandile F. Tsabedze, Caroll Hermann, Thabile S. Manengela

**Affiliations:** 1 Community Psychosocial Research, North-West University, Mafikeng 2745, South Africa; dineojeniffermelamu@gmail.com (D.J.M.); 29595487@nwu.ac.za (T.S.M.); 2 Department of Psychology, University of South Africa, Pretoria 0003, South Africa; 3 Optentia Research Unit, North-West University, Mafikeng 2745, South Africa; caroll.hermann@nwu.ac.za

**Keywords:** COVID-19, psycho-social well-being, rural youth, lockdown, mental health

## Abstract

The COVID-19 pandemic and its associated lockdowns had widespread psycho-social implications globally. However, the unique experiences of young adults in under-researched rural communities, such as Lonely Park in South Africa’s North West Province, remain poorly documented. This study explores the psycho-social well-being of young adults aged 18–24 in the Lonely Park community during the national COVID-19 lockdown from 23 March 2020 to 30 April 2020, with a particular focus on their emotional, psychological, and social experiences. Using a qualitative, phenomenological design rooted in Bronfenbrenner’s Ecological Systems Theory (EST), data were collected from 14 purposively sampled participants through two focus group discussions. Thematic analysis was conducted following Braun and Clarke’s six-phase framework. Ethical clearance was obtained from the Health Research Ethics Committee, and participants provided informed consent. Two main themes emerged, (1) psychological and (2) social well-being. Six sub-themes were identified: (1) negative emotions linked to lockdown, (2) fear of COVID-19 and its consequences, (3) rumination and anxiety, (4) disruption of social and educational routines, (5) coping strategies, and (6) structural limitations of healthcare and support systems. Participants experienced intense fear, boredom, isolation, and a sense of helplessness. Yet, coping mechanisms such as communication, spirituality, exercise, and adherence to public health regulations provided resilience. Some reported depression and financial strain due to job loss and school disruptions. The findings highlight the complex interplay of psychological, emotional, and social stressors in rural young adults during the pandemic. Policymakers and mental health practitioners must prioritise targeted psycho-social interventions for youth in under-resourced rural settings. A call is made for culturally responsive mental health programmes that incorporate local voices, especially in rural African contexts.

## 1. Introduction

The emergence of the COVID-19 pandemic in late 2019 marked one of the most disruptive global health crises in modern history. The disease, caused by the SARS-CoV-2 virus, was declared a global pandemic by the World Health Organization (WHO) [1,2] on 11 March 2020. Governments worldwide implemented drastic measures, including nationwide lockdowns, to contain the virus. These restrictions, while effective in slowing the transmission, profoundly altered everyday life, especially for young people navigating critical developmental transitions.

Young adulthood (ages 18–24), as framed by Erikson’s psychosocial theory [3], represents a formative stage characterised by identity formation, social exploration, and vocational development. The sudden imposition of social distancing, the suspension of academic activities, job losses, and isolation disrupted these milestones. This psychosocial disruption was even more pronounced in rural contexts where systemic neglect, under-resourced infrastructure, and limited digital access compounded the psychological burden [4].

To manage the spread of the virus, South Africa implemented one of the strictest lockdowns globally, beginning on 23 March 2020 [5] till 30 April 2020. This included strict limitations on movement, business operations, social gatherings, and access to public spaces. With each alert level, restrictions were gradually eased—from total closure under Level 5 to limited social activity under Level 1 [6].

During this period, young adults faced considerable hardship. Many lost jobs, experienced relationship breakdowns, and mourned the deaths of family and peers. The lockdown intensified pre-existing social and economic vulnerabilities, including limited access to mental health services. Cultural variations in responses to the pandemic further shaped how communities reacted to lockdown measures [7]. Participants expressed psychological distress related to the fear of losing loved ones, job insecurity, and prolonged isolation. Common coping strategies included adhering to safety guidelines, praying, exercising, maintaining communication with others, and resting [8,9].

A review of the existing literature revealed no studies specifically addressing the psycho-social experiences of young adults in the Lonely Park, North West Province region. This gap highlights the importance of research in under-resourced rural areas, particularly as Mafikeng, under which Lonely Park falls, reported a high number of COVID-19 cases compared to other local municipalities [10]. The local clinics lacked essential resources such as personal protective equipment, soap, water, and adequate staff during lockdown, further compounding the crisis [11,12,13]. Rural communities continue to be neglected in both research and policy interventions [14], and decisions affecting these areas are often made without sufficient input from local stakeholders.

This study was conducted in Lonely Park, a semi-rural area in Mafikeng, North West Province, South Africa. It aimed to explore how young adults in this under-researched setting experienced the COVID-19 lockdown, particularly its impact on their emotional, psychological, and social well-being.

Bronfenbrenner’s Ecological Systems Theory (EST) [15] provides the conceptual lens for this study. EST posits that an individual’s development is influenced by nested environmental systems: the microsystem, mesosystem, exosystem, macrosystem, and chronosystem.

EST thus allows for a multi-layered interpretation of young adults’ psycho-social well-being during the COVID-19 lockdown in a rural African context.

## 2. Materials and Methods

A qualitative phenomenological design was adopted to capture the lived experiences of young adults during lockdown. This inductive approach is appropriate for exploring emotional and psychological processes grounded in real-world settings.

The study was conducted in Lonely Park, Mafikeng, a rural, underdeveloped area characterised by limited healthcare infrastructure, digital exclusion, and high youth unemployment. According to Statistics South Africa [16], Lonely Park has approximately 1001 residents. The dominant language spoken is Setswana (81%), followed by Sesotho (4%), isiZulu (3%), isiXhosa (3%), and Shona (9%). Participants had to reside in Lonely Park, Mahikeng, between the ages of 18 and 24 and be fluent in English or Setswana. There were no other exclusion criteria.

Purposive sampling was used to recruit 14 participants (5 males and 9 females) aged 18–24. The semi-structured interviews were conducted individually to ensure privacy and confidentiality in a community hall in Lonely Park in English and Setswana, audio-recorded, and transcribed verbatim. Several questions guiding the discussion were asked, i.e., “*How did you feel when you first heard about the COVID-19 lockdown?*”; “*Has the pandemic caused worries about your future?*” and “*How are the COVID-19 lockdown related worries different from the worries you had before?*”.

The researcher ensured credibility and trustworthiness through member-checking for verification and feedback, validating interpretations, persistent observation and triangulation by the research assistant present during discussions, and the use of direct quotations.

Braun and Clarke’s [17] six-phase thematic analysis method was used. This involved familiarisation, generating initial codes, searching for themes, reviewing themes, defining and naming themes, and producing the final report. Codes were compared, refined, and grouped based on their similarities and differences. Themes and sub-themes were then developed by examining the relationships between codes. The themes were reviewed for coherence and adjusted to ensure clear distinctions. The researcher ensured that the final themes accurately represented the full dataset by revisiting the transcripts in two phases. Each theme was defined, named, and supported by participant narratives.

The study received ethical approval from the Health Research Ethics Committee (NWU-HREC) and complied with South African ethical guidelines for human research. Participants were informed of potential risks, provided with psychologist referrals, and offered post-study debriefing. Participants provided written informed consent and agreed to a non-disclosure policy. Data are stored in a password-protected folder at the NWU and available on request.

## 3. Results

Participants came from diverse educational backgrounds ranging from Grade 10 to postgraduate degrees. Recruitment was facilitated via local advertisements and gatekeeper permission from tribal authorities. The sample included five (36%) males and nine (64%) females. Participants’ ages ranged from 18 to 24 years with a mean age of 20 and an SD of 2.1.

Educational backgrounds varied. One participant had completed Grade 10, two had completed Grade 11, six had completed Grade 12, two held higher certificates, one held a diploma, one a bachelor’s degree, and one an honours degree. (See Table 1 below).

Two main themes and six sub-themes emerged from the data, with associated sub-categories. Select quotations (translated where necessary) illustrate participant voices.

### 3.1. Theme 1: Negative Emotions Related to Lockdown

Participants reported overwhelming boredom, loneliness, sadness, and emotional distress.

*“First time when I heard about COVID-19 lockdown, I was so scared, mixed emotions at the same time. I was worried about how we are going to live our live our lives to the fullest because we were worried about what we were going to eat and what to wear and when the disease is going to end”.*

(Group 2, Participant 1)

*“Coughing… the first time when I heard about the COVID-19 lockdown, I felt frustrated because I was always over-thinking about what is going to happen to my school studies, whereas ne re phela re prohibitiwa goya sekolong (we were not allowed to go to school) until further notice. Moreover, ke gore re saletse ko morago (we were left behind) and many of us failed so COVID-19 ere affectile fao (affected us there)”.*

(Group 2, Participant 4)

*“Most of the time o dula ole one (you were sitting alone), nna ke modumo, ke rata go gowa batho (I am noisy, I like to call people) even if ke tlaba itsi gore ketla (when I am coming they know), I like to touch people and during COVID-19 lockdown we were not allowed gore re tshware bathole go bua le batho face-to-face so neke feel lonely (to touch people even to talk to them face-to-face so I felt lonely)”.*

(Group 1, Participant 4)

*“I was anxious because neke tshaba gore ketla lose bagolo baka ba tshwereke COVID-19 (I was afraid that I could lose my parents due to COVID-19) and gape kene nagana gore le nna botla ntshwara gantse keya sekolong ka gore bo tsena in social gathering and gape ko sekolong nere phela rele bantsi (I thought I will get infected when going to school because it affects an individual on social gatherings and at school, we are always many”.*

(Group 2, Participant 5)

### 3.2. Theme 2: Fear of COVID-19

Fear was a common emotional response during the COVID-19 pandemic, driven largely by misinformation and a lack of clear public knowledge.

Sub-themes included the following:

- Fear of losing loved ones due to high mortality rates.

*“Bolwetsi bojwa Corona before botsena neke sena matshenego a gore like ne kesa nkeke nagana sepe ka loso but after bofetsa go introducega (Before COVID-19 was introduced, I did not worry about death but after it was introduced), I was always worried gore what if keya go lose motsadi wame”.*

(I lose my parent) (Group 1, Participant 3)

*“Mmm…I was always worried because I was afraid that I could lose my family members, friends and colleagues at work”.*

(Group 1, Participant 5)

*“Le bakalegolo la go baka matshwenyego ame ene ele bagolo bame gonne bolwetsi bo ne bo rata go tshwara bagolo baba godileng (The main reason that caused my worry was my parents because the disease was likely to attack old people)”.*

(Group 2, Participant 5)

*“I was worried gore keya go lose batho bake baratang (because I could lose the people I love) the most, which is my family”.*

(Group 1, Participant 6)

- Fear of infection from essential activities like grocery shopping or attending school.

*“Mmm… I was always worrying about getting COVID-19”.*

(Group 2, Participant 6)

*“COVID-19 e damagitse tlhaloganyoyaka I was always worrying about being infected with COVID-19, lefa ne bafouna ko gae ne setseke nagana gore motho wako gae o nale COVID-19 or o tlhokafetse because of COVID-19 (COVID-19 damaged my thinking even if they were calling at home what I was thinking was that one member is having COVID-19 or had passed away due to COVID-19)”.*

(Group 2, Participant 7)

- Worry about financial provision after job losses.

*“Kwa company enengke e direlaeleng gore ke Rebawetse general dealer bane ba retrencher bathoand le nna I was also part of people retrenched seo se dirileresafepa our family members le gore resapatela le dikoloto nyana tseneng redi dirile (At the company that I was working for which is Rebawetswe general dealer, people were retrenched, and I was part of them. That made me to be unable to provide for my family and not being able to settle debts)”.*

(Group 1, Participant 6)

*“Uhhhm… (I ended up losing my job because I was working at a cinema) nna ke felleditse ke feletswe ke mmereko because nekebereka ko cinema, so we lost customers because batho basa kgone go tsena bale bantsi so re feleditse e tswala (people did not come so it ended up being closed) and everything was shut down. I could not survive, I could not provide for my kid and my family because I am a breadwinner to them, I was worried about how we will survive”.*

(Group 2, Participant 1)

*“I was worried because I lost my job so there will not be any source of income at home”.*

(Group 2, Participant 3)

### 3.3. Theme 3: Psychological Distress and Rumination

This theme reflects participants’ reports of increased rumination and anxiety, largely driven by misinformation about COVID-19.

Participants experienced the following:

- Rumination due to a lack of credible information.

*“Lack of information affected me in a bad way because it made me to over-think about who is going to die when and how”.*

(Group 1, Participant 2)

*“E affectile (it affected me) because I was over-thinking. It turned gore go tlile go introduce’iwa vaccine (that the vaccine will be introduced) batho ba bangwe resa itsi (some of us did not know)”.*

(Group 2, Participant 2)

*“Lack of information affected my psycho-social well-being because it made me to always over-think”.*

(Group 2, Participant 5)

- Anxiety from vaccine misinformation.

*“I was anxious because I did not have most of the information about the disease and vaccine and people came up with many stories about the vaccine … that if you vaccinate, you will die or not be able to have kids”.*

(Group 1, Participant 3)

*“…re feleditse re kreiya le wrong information ya vaccination (we ended up getting wrong information about the vaccine)”.*

(Group 1, Participant 4)

- Participants stated that COVID-19’s influence on their psycho-social well-being included worrying them about adjusting to new routines. They also experienced depression.

*“Experience yaka e nnile (my experience was to) worry and fear to adjust to new routines actually financially, employment insecurity le lack of excising and loss of normal routines”.*

(Group 2, Participant 2)

*“To emphasise on what has been said, ne kesakgone go phela (I was not used to live) the way ketlwaetseng (I used to)”.*

(Group 2, Participant 3)

*“I was always having ups and downs because I was having anxiety, depression and I was over-thinking time and again about COVID-19”.*

(Group 1, Participant 3)

*“We didn’t cope; we had depression, some of us re feleletse baitiretse bojalwa gwa nna (ended up doing our own alcohol)”.*

(Group 1, Participant 4)

*“Di tarvern nedi tswetse nere reka kadi back door not knowing the disadvantage ba bojwala boo gore bo mixitswe ka eng and jang (taverns were closed, we bought beer using back doors not knowing the disadvantage of that alcohol and was used to manufacture it)”.*

(Group 2, Participant 5)

### 3.4. Theme 4: Disruption of Social Life and Education

Many struggled with academic delays, digital exclusion, and peer disconnection.

*“Yes, because it affected my psycho-social well-being in a sense that it affected my emotions towards people. I was not happy and comfortable to be around people”.*

(Group 1, Participant 4)

*“Yes, because every single day neke nagana (I was thinking) only negative things not positive things nere nagana bo (I was thinking) what if, what if ke tsamaya ko ntle mapodisa ba ntshwara ka 12am (I am walking around 12a.m. then the police catch me) … We were wondering gotla di ragalaeng re phela ka di (what will happen when we live with) negative thoughts”.*

(Group 1, Participant 5)

*“Eeeh (Yes) I think COVID-19 affected my psycho-social well-being only because I was living with anxiety and I also felt bored and lonely during the pandemic, which affected my mental state as I was always over-thinking”.*

(Group 2, Participant 6)

*“COVID-19 did not affect my psycho-social well-being only because I lost my job and that made me realise that it was because of COVID-19 lockdown”.*

(Group 1, Participant 2)

*“No, because it led me to poverty as I lost my job due to people being retrenched from work”.*

(Group 1, Participant 5)

*“No, I do not think so because COVID-19 e ntlogetse ke nale (left me with) mental breakdown because it ended up taking my family members”.*

(Group 2, Participant 4)

### 3.5. Theme 5: Coping Strategies

Participants engaged in several coping strategies to overcome fears and concerns. Most people adhered to COVID-19 protocols by wearing masks, sanitising, and social distancing.

*“I was wearing a mask each and every day and I was sanitizing for at least 20 seconds; I was staying indoors”.*

(Group 1, Participant 3)

*“Social distancing, always washing hands, trying go dula mo ntlong (to stay in the house) each and every day, to eat healthy food and re saje dijo tse ditsididi (not eating cold food). Trying to keep our bodies warm gore resa kreiya serame (so that we cannot get cold)”.*

(Group 1, Participant 5)

Participants used prayer and other spiritual activities as coping strategies during COVID-19.

*“I was always praying that this pandemic can end until God decided to hear my prayers and end this disease”.*

(Group 1, Participant 3)

*“I was always praying because I was unable to go to church”.*

(Group 2, Participant 3)

Most participants claimed to either sleep or do physical exercise as a coping mechanism.

*“Neke (I was) exercise (exercising) each and every day so that I can get my mind out of this COVID-19 and even though neke exercise mo ntlong (I was exercising in the house) because nego sena chance yabo goya gym because ne ditswetse by that time (there was no chance for gym because they were closed by that time)”.*

(Group 2, Participant 3)

*“Go deal ka COVID-19 neke phela ke itlhokomela ke exercise ke robala enough (In order to deal with COVID-19 lockdown, I was always protecting myself by exercising and getting enough sleep)”.*

(Group 1, Participant 4)

All participants valued their communication channels with friends and family, mostly via phone during the pandemic.

*“I was calling my family members to check whether they were okay or not so that made me to feel no more worried”.*

(Group 1, Participant 5)

### 3.6. Theme 6: Systemic and Structural Constraints

Participants described inadequate rural healthcare (no PPE, soap, water), long clinic queues, and absent psychological support structures.

*“Our clinic didn’t even have gloves or masks. The line was always long, and I was scared to go there.”.*

*“I was associating with other people ka phone (with a phone) and video call my family kelekgakala and ledi neighbours (when I am far and also neighbours)”.*

(Group 1, Participant 7)

## 4. Discussion

The findings underscore how the COVID-19 lockdown severely disrupted the psycho-social equilibrium of young adults in rural South Africa. Two main themes (psychological and social well-being) emerged, and six subthemes were identified.

### 4.1. Theme 1: Negative Emotions Related to Lockdown

Participants reported overwhelming boredom, loneliness, sadness, and emotional distress. Many described the sudden loss of routine and social interaction as emotionally destabilising. The implementation of social distancing during the COVID-19 lockdown contributed significantly to increased levels of anxiety and depressive symptoms, particularly among young adults. Studies have shown that the prevalence of these symptoms more than doubled compared to pre-pandemic levels [18]. Participants who adhered to distancing measures reported feeling isolated, bored, and emotionally neglected feelings exacerbated by the disruption of familiar social practices such as traditional gatherings and funerals [5,13,19].

The findings from this study reflect similar trends. Participants described feeling lonely, anxious, and emotionally drained. Their inability to participate in everyday social activities—such as playing soccer, meeting friends, or spending time with loved ones—intensified their sense of isolation and fear. Many avoided social interactions out of concern for transmitting or contracting the virus [9,19].

Participants reported a surge of negative emotions upon learning about the lockdown. This included anxiety, worry, and boredom, all of which negatively affected their psycho-social well-being. These reactions reflected the sudden disruption in personal routines and social roles that are critical to identity formation and exploration in the emerging adulthood phase [18], with an overwhelming fear of the loss of income and job opportunities. Yeo’s [19] study of young adults in Singapore concurs with the findings of the Lonely Park study.

Several participants mentioned “over-thinking” (rumination) about the pandemic, which further contributed to emotional distress. The existing literature supports these experiences, showing that the lockdown period significantly impacted young adults’ mental and emotional health [8,20]. Anxiety was often heightened by news of rising death rates and the general uncertainty surrounding the pandemic. Other studies found that mindfulness counteracted the impact on psychosocial well-being [20,21].

### 4.2. Theme 2: Fear of COVID-19

In a U.S. study with Latinx participants, many expressed fear stemming from inconsistent and unreliable information about safety measures and available resources [21]. When a health crisis emerges, fear is often an immediate psychological reaction. However, when prolonged, it can aggravate existing mental health conditions and increase psychological distress [22].

The pandemic, along with public health interventions such as lockdowns, led to significant disruptions in daily life. Job losses, financial insecurity, and isolation have had a lasting impact on young adults’ mental well-being [23]. Elevated levels of stress, compulsive behaviours, and mood disturbances have all been associated with fear of the virus [24]. Persistent worry about contracting COVID-19, infecting loved ones, or spreading the virus to others added to the psychological strain [25].

Findings from this study align with existing research. Participants reported deep concern during the lockdown, especially the fear of losing family members, friends, or colleagues. This fear, coupled with loneliness and poor psychological well-being, led to anxiety and, in some cases, self-diagnosed depression. Participants’ experiences echoed broader psychological responses to the pandemic, including anxiety, depression, grief, anger, guilt, social withdrawal, and post-traumatic stress [20].

### 4.3. Theme 3: Psychological Distress and Rumination

Many young adults were unable to attend school regularly, which affected their academic performance and caused distress. This disruption had a negative impact on their psycho-social well-being, as they expressed ongoing concern about their education [4]. In addition, their relationships with friends were strained. The lockdown limited opportunities for regular communication and social interaction, altering the way they connected with peers.

Several studies found that ruminant thoughts and the perception of vulnerability had a positive relationship [20,22,26], causing psychological maladjustment and cognitive discomfort. Rumination acted as a detrimental factor to psychological well-being by reinforcing negative thought patterns.

However, a Dutch study [23] found that females, older individuals, and people with lower education levels were more likely to ruminate about COVID-19.

### 4.4. Theme 4: Disruption of Social Life and Education

During the COVID-19 pandemic, emerging adults experienced a significant decline in life satisfaction and mental health well-being [23,24]. The pandemic disrupted certain developmental trajectories that necessitated significant adjustments and coping strategies. Sorgente [25] claimed that family and peer support can help emerging adults face adversities. Goldstein [27] found that the increased time spent with their mothers was seen as a challenge and an opportunity to build stronger relationships.

Participants were isolated from their peers, together with fears that the quality of their education would suffer due to online classes and an uncertain routine [26,27]. Yeo [19] found that the pandemic not only disrupted education but also internships. This was evident in text messages regarding the instability about future work and education.

### 4.5. Theme 5: Coping Strategies

Eloff [28] found that in South Africa, emerging adults relied heavily on spiritual (strategies) to manage stress, social distancing (relationship building), and online learning (education). The study also found that new skills were developed and that they engaged in structured activities. Additionally, participants showed resourcefulness in combining multiple sources, such as music, family, and self-care practices, like prayer.

### 4.6. Theme 6: Systemic and Structural Constraints

In line with Bronfenbrenner’s model [15], as seen in Table 1 above, family bonds (microsystem) were stressed due to fears of infection, job loss, and intra-household tensions [27]. Educational and peer-based connections (mesosystem) were broken, resulting in academic decline and social isolation [26].

Weak health services and insufficient community support (exosystem) exacerbated psychological distress [26,27]. Economic inequalities and cultural misunderstandings (macrosystem) about the COVID-19 vaccination played a critical role. The temporal impact of the lockdown (chronosystem), lasting more than a year, compounded cumulative stress and uncertainty.

These results mirror international studies on rural youth [4,7,29,30], but uniquely highlight the combined influence of rurality, poverty, and digital exclusion. Moreover, cultural dimensions (e.g., reliance on prayer, collectivism) shaped coping behaviours differently from youth in urban or Western contexts [11,12,13,14].

## 5. Conclusions

Young adults experienced fear in various forms—fear of death, fear of losing loved ones, and fear of the virus itself. These fears contributed to heightened anxiety, uncertainty, and symptoms of depression. In remote or semi-rural communities such as Lonely Park, healthcare concerns often provoke greater distress due to limited access to medical services. As a result, such communities are especially vulnerable during public health crises like COVID-19.

The strengths of the study, lies in the rich, firsthand data from young adults in a rural town. The methodology is rigorous and followed ethical guidelines and validated analytical frameworks. The limitation of the study is the small size, which limits generalizability, and the self-reported mental health symptoms were not clinically verified.

The findings indicate that the psycho-social well-being of young people in this context was significantly affected. Many lived in a constant state of fear and emotional disruption.

It is recommended that national, provincial, and local governments prioritise the healthcare needs of disadvantaged communities, with particular attention to young people. Strengthening healthcare infrastructure and providing accessible mental health services is essential. Collaborative, youth-focused care is necessary to ensure that their developmental and psychological needs are addressed effectively. Further, it is recommended that tailored, multilingual public health literacy campaigns are implemented to counter misinformation. Sustainable resilient support groups are to be established in rural communities to negate the impact of future pandemics.

## 6. Strengths and Limitations of the Study

The study investigates the research gap of studying the under-researched psychosocial well-being of emerging adults in the rural North West Province in South Africa, which has received minimal empirical attention. Anchoring the study in Bronfenbrenner’s Ecological Systems Theory strengthens the analytical depth and ensures that the results are interpreted beyond individual-level phenomena. The phenomenological design enabled the researcher to uncover subjective experiences, thereby understanding psych-social constructs during the pandemic.

Due to the qualitative and purposive sampling strategy nature of the study, within a single rural location, the findings are not generalizable to other South African rural communities. The study is small and limited in diversity and may constrain perspectives. Further, the study relied on self-diagnosed symptoms without formal clinical assessment tools. Due to social undesirability bias, some participants might have underreported information around perceived sensitive information. Moreover, language and translation constraints may have introduced minor semantic inaccuracies or the loss of cultural meaning in certain quotations. The scope for future studies should include a longitudinal approach for more dynamic insights into coping and recovery.

Despite its limitations, this study provides valuable, in-depth insights into how rural South African youth experienced the COVID-19 lockdown.

## Figures and Tables

**Table 1:** Bronfenbrenner’s Ecological Systems Theory and the impact of the COVID-19 pandemic.

EST	System	Impact
Microsystem	Family, school, peers	Direct impact due to physical distancing
Mesosystem	Links between micro-contexts	Weakened, especially in rural areas
Exosystem	Healthcare infrastructure	Became less accessible
Macrosystem	Socio-political structures and cultural norms	Shaped compliance with regulations
Chronosystem	Historical events (such as the COVID-19 pandemic)	Framed temporal disruption.

## Data Availability

Deidentified data are available on request.
